# Impact of antibiotic-impregnated catheters on the reduction in operations for cerebrospinal fluid shunt infection since 1995: evidence from the UK Shunt Registry

**DOI:** 10.1186/2045-8118-12-S1-O9

**Published:** 2015-09-18

**Authors:** John D Pickard, Hugh K Richards, Helen M Seeley

**Affiliations:** 1 University of Cambridge, UK

## Introduction

Various methods have been introduced to reduce CSF shunt infection including institutional protocols, antibiotic prophylaxis and antibiotic-impregnated catheters. Have such measures led to a sustainable reduction in CSF shunt infections?

## Methods

The United Kingdom Shunt Registry is a paper-based reporting system for CSF shunts inserted in the 34 UK neurosurgery units since May 1995. This Report is based on data downloaded on 23rd January 2015 from the master database that gave a shunt procedure dataset from 1st January 1995 to 31st December 2014 of 53,767 procedures in 29,341 patients. The infection risk was calculated as the proportion of procedures subsequently revised for infection based on “intention to treat” recorded at the time of surgery where the follow-up was greater than nine months. Subsequent bacteriological confirmation was not available.

## Results

There was a trend towards a fall in shunt infection risk in both adults and children over recent years. With regard to the impact of the introduction of antibiotic-impregnated catheters, our previously published study was based on a cohort of 994 pairs matched for age, diagnosis, number of previous procedures and gender procedures recruited up until the end of 2006. The infection risk in that cohort was reduced from 4.7% using conventional catheters to 3.0% using Bactiseal catheters. Data from 2007 onwards was used to construct a second matched-pair comparison. 11938 procedures were identified where patients could be defined by age, diagnosis gender and number of previous revisions. 6302 antibiotic-impregnated catheters and 5636 conventional catheters were used. This data set yielded 4011 matched pairs. The calculated infection risk was 1.87% in conventional catheters and 1.12% in antibiotic-impregnated catheters (p=0.006).

## Conclusions

The overall risk of shunt infection at all ages has reduced over recent years. Antibiotic-impregnated catheters have significantly reduced shunt infections but other factors may have played a role.
